# Metabolic control of induced pluripotency

**DOI:** 10.3389/fcell.2023.1328522

**Published:** 2024-01-11

**Authors:** Sergey A. Sinenko, Alexey N. Tomilin

**Affiliations:** Institute of Cytology, Russian Academy of Sciences, Saint-Petersburg, Russia

**Keywords:** induced pluripotent stem cells (iPSCs), embryonic stem cells (ESCs), cellular reprogramming, pluripotency, mitochondria, oxidative phosphorylation (OxPhos), glycolysis, reactive oxygen species (ROS)

## Abstract

Pluripotent stem cells of the mammalian epiblast and their cultured counterparts—embryonic stem cells (ESCs) and epiblast stem cells (EpiSCs)—have the capacity to differentiate in all cell types of adult organisms. An artificial process of reactivation of the pluripotency program in terminally differentiated cells was established in 2006, which allowed for the generation of induced pluripotent stem cells (iPSCs). This iPSC technology has become an invaluable tool in investigating the molecular mechanisms of human diseases and therapeutic drug development, and it also holds tremendous promise for iPSC applications in regenerative medicine. Since the process of induced reprogramming of differentiated cells to a pluripotent state was discovered, many questions about the molecular mechanisms involved in this process have been clarified. Studies conducted over the past 2 decades have established that metabolic pathways and retrograde mitochondrial signals are involved in the regulation of various aspects of stem cell biology, including differentiation, pluripotency acquisition, and maintenance. During the reprogramming process, cells undergo major transformations, progressing through three distinct stages that are regulated by different signaling pathways, transcription factor networks, and inputs from metabolic pathways. Among the main metabolic features of this process, representing a switch from the dominance of oxidative phosphorylation to aerobic glycolysis and anabolic processes, are many critical stage-specific metabolic signals that control the path of differentiated cells toward a pluripotent state. In this review, we discuss the achievements in the current understanding of the molecular mechanisms of processes controlled by metabolic pathways, and vice versa, during the reprogramming process.

## Introduction

Epiblast cells residing in the inner cell mass (ICM) of the preimplantation embryo are pluripotent stem cells that possess the capacity to differentiate into all cell types of the mammalian organism, except extraembryonic trophoblast and primitive endoderm. Epiblast cells under specific culture conditions are capable of infinitely maintaining a pluripotent state *ex vivo* and are referred to as embryonic stem cells, or ESCs (Robertson et al., 1986; Thompson et al., 1989; Thomson et al., 1998). The breakthrough studies of Shinya Yamanaka’s laboratory discovered in 2006 an important process of reactivation of the pluripotency program in terminally differentiated cells, establishing induced pluripotent stem cells (iPSCs) via ectopic expression of the transcription factors (TFs) OCT4, SOX2, KLF4, and cMYC (OSKM) (Takahashi and Yamanaka, 2006). Thus, iPSCs are artificially generated counterparts of ESCs, sharing all of their main features. Along with OSKM-based methods, somatic cells can be reprogrammed to pluripotency by chemical treatment (Hou et al., 2013; Liuyang et al., 2023). Nowadays, iPSC technology has become an invaluable tool to investigate the molecular mechanisms of cell differentiation and embryonic development, to model human diseases *ex vivo*, and to develop therapeutic drugs for their treatment. Also, iPSCs hold tremendous promise in cell replacement therapy and regenerative medicine (Di Giorgio et al., 2007; Karagiannis et al., 2019; Yamanaka, 2020; Sinenko et al., 2021a; Herriges et al., 2023). iPSC reprogramming becomes a unique experimental system to investigate molecular mechanisms that govern the reverse cell fate transition. It also provides important clues to understand the molecular mechanisms of natural cell fate specification during mammalian development. Due to reprogramming to pluripotency, cells undergo dramatic changes in various cellular processes, including signal transduction and transcription networks, metabolic flux regulation, and retrograde signaling of mitochondria to the nucleus (Prieto et al., 2020).

Recent studies have identified molecular features of iPSC reprogramming via various genetic and genomic approaches. Cooperative binding of TFs, along with their transient and permanent interactions with chromatin factors, orchestrate this reprogramming (Chen et al., 2016; Chronis et al., 2017; Knaupp et al., 2017; Li et al., 2017). Poised iPSC intermediates and their molecular markers have also been identified during cellular reprogramming (Schwarz et al., 2018; Ha et al., 2022). Single-cell analyses determined the checkpoints and cell fate decisions of this process (Zunder et al., 2015; Tran et al., 2019a; Francesconi et al., 2019; Guo et al., 2019). In general, reprogramming is characterized by the silencing of the somatic gene expression program and the activation of the pluripotency gene network. Alongside master regulators of transcription, epigenetic modifiers play a crucial role in pluripotency acquisition. With the help of the above approaches, it has also been established that fibroblasts undergo mesenchymal-to-epithelial transition, progressing through primitive streak-like intermediates, resulting in only a small part of cells becoming mature iPSCs (Takahashi et al., 2014; Parenti et al., 2016; Schiebinger et al., 2019; Liu et al., 2020; Xing et al., 2020). Molecular marker expression analysis allowed to establish at least three stages of the iPSC generation process: early (initiation), mid (maturation), and late (stabilization) stages (Samavarchi-Tehrani et al., 2010; Apostolou and Hochedlinger, 2013; Ha et al., 2022). Cells undergoing reprogramming at each of these stages have unique gene expression and metabolic profiles. Proliferation and suppression of proapoptotic and senescent genes, including p53, during the early stages (days 1–4) of the process are critical for achieving efficient reprogramming to pluripotency (Schiebinger et al., 2019). The early stage of iPSC reprogramming is the most important stage at which many dramatic molecular and cellular events controlling metabolic pathways, redox events, induction of oxidative burst, mitochondria remodeling, mitophagy, induction of innate immunity pathway, and epigenetic landscape occur (Hansson et al., 2012; Kida et al., 2015). The intermediate stage is less investigated in terms of metabolic changes, while the late stage of reprogramming is characterized by almost complete activation of the pluripotent factor network, increased proliferation, dominance of glycolytic metabolism, and extensive epigenetic transformation. Activating glycolytic flux during iPSC generation improves the efficiency of the process, while impeding glycolysis has a suppressive effect (Folmes et al., 2011; Panopoulos et al., 2012; Zhang et al., 2012). Changes in the expression of metabolic genes, which underlay the shift from oxidative phosphorylation (OxPhos) to glycolysis, precede the activation of the pluripotency circuitry (Folmes et al., 2011; Hansson et al., 2012; Mathieu et al., 2014; Prigione et al., 2014; Cacchiarelli et al., 2015). In this review, we discuss recent achievements in the study of interactions among the factors involved in the induction of pluripotency with metabolic pathways at the levels of 1) regulation of metabolic genes, 2) regulation of mitochondria function and biogenesis, 3) mitochondria retrograde signaling, and 4) reactive oxygen species (ROS) signaling. We also discuss the importance of mitochondria and OxPhos for successful cell reprogramming. Understanding how molecular networks that regulate cellular reprogramming interact with metabolic processes would help to develop more efficient and safe iPSC generation protocols, meeting the demands of regenerative medicine.

## Main characteristics of the stages of reprogramming toward pluripotency

**FIGURE 1:**
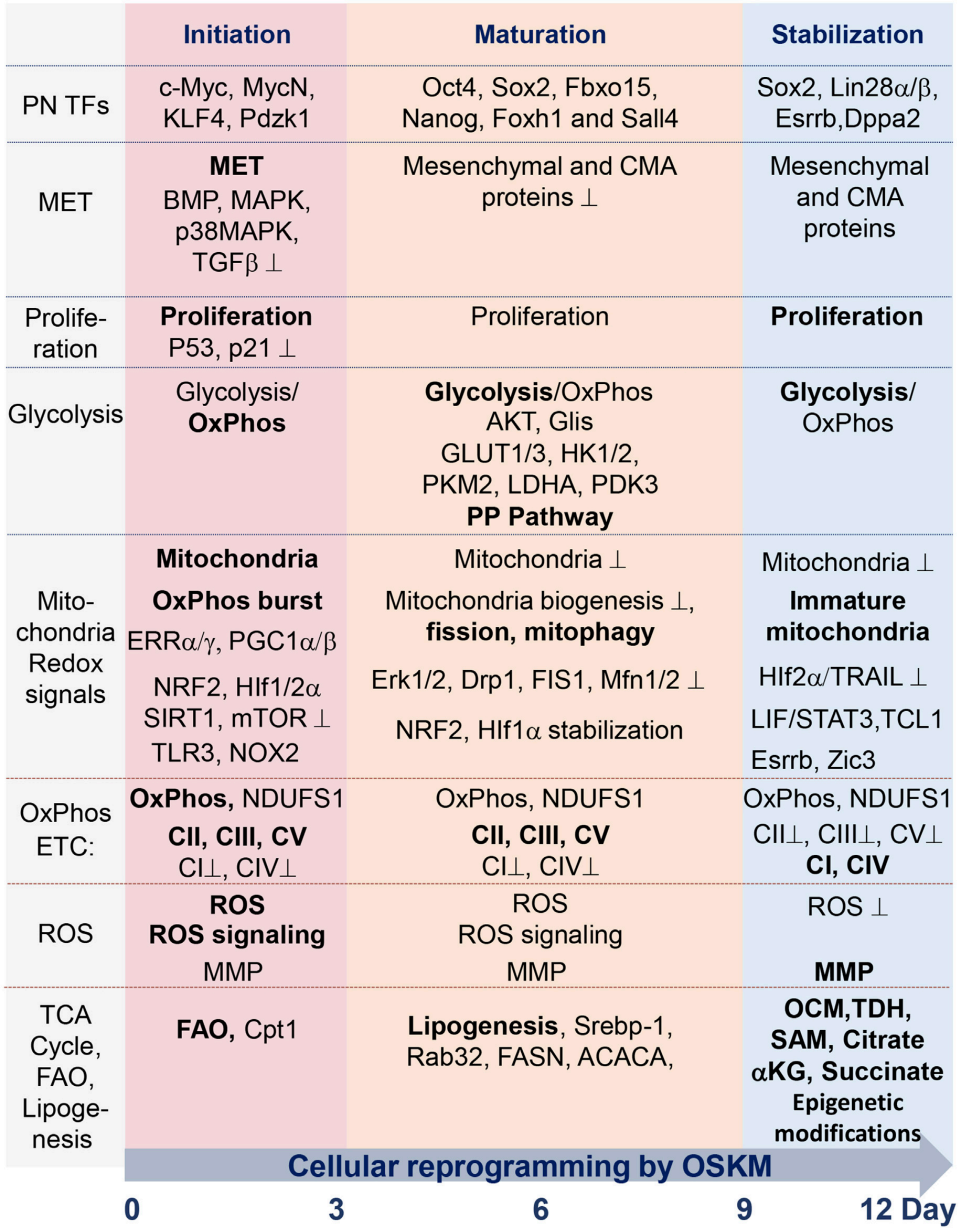
Summary table of molecular and metabolic characteristics of cells at different stages of reprogramming toward pluripotency state, mediated by ectopic expression of TFs Oct4, Sox2, Klf4, and c-Myc (OSKM). The process occurs during the sequential initiation, maturation, and stabilization stages, all taking place within a 12-day time interval. Critical factors, the functions of which are required for the reprogramming process, are indicated in regular font. The dominance of factors or processes is marked in bold. Functions and factors that are downregulated during reprogramming are marked with a suppression sign (⊥); see details in the text and abbreviations.

To date, two approaches to reprogramming terminally differentiated cells into a pluripotent state have been established. One makes use of reprogramming TFs (Takahashi and Yamanaka, 2006), while the other, which was developed more recently, uses a cocktail of several low-molecular-weight inhibitors and activators (Hou et al., 2013). These approaches appear to be quite different in terms of the molecular mechanisms, staging, timing, and epigenetics of reprogramming, and a chemical-based approach has been practically uninvestigated in terms of its metabolic regulation [reviewed in (Meir and Li, 2021; Lange et al., 2022; Wang et al., 2023)]. Here, we discuss mainly the current view of the metabolic characteristics described for reprogramming from terminally differentiated somatic cells to pluripotent stem cells using the transgene-based strategy. Four TFs, Oct4, Sox2, Klf4, and cMyc, convert the epigenome of somatic cells into an ESC-like pluripotent state. This approach has been extensively investigated in terms of the molecular mechanisms of its regulation by transcription factor network, metabolic, and epigenetic factors (Buganim et al., 2013; Takahashi and Yamanaka, 2016; Deng et al., 2021; Teslaa and Teitell, 2015; Wu et al., 2016; Nishimura et al., 2019; Tsogtbaatar et al., 2020; Meir and Li, 2021). Stage-dependent landmarks of cell reprogramming have been identified in several studies through genome-wide analyses of the transcriptome, proteome, epigenome, and metabolome (Maherali et al., 2007; Mikkelsen et al., 2008; Zhao et al., 2009; Stadtfeld et al., 2010; Gundry et al., 2012; Hansson et al., 2012). A dramatic reconfiguration of the proteome during the first and last 3 days of the 2-week interval of mouse cell reprogramming has been uncovered. Between these resetting phases (i.e., during the intermediate phase) more moderate proteome changes occur (Hansson et al., 2012). Analyses revealed a high coordination of expression of functionally linked proteins during the reprogramming phases. Genome-wide transcriptome analysis also revealed two similar transcriptional waves (Polo et al., 2012). These data, along with the results of single-cell expression analyses (Buganim et al., 2012), suggest that iPSC formation is a stepwise process tightly controlled by a set of functionally linked genes. Based on the revealed stage-specific regulatory networks, three phases of the reprogramming process were identified: initiation, maturation, and stabilization (Figure 1) (Mikkelsen et al., 2008; Samavarchi-Tehrani et al., 2010; Buganim et al., 2012; David and Polo, 2014; Meir and Li, 2021). During the initiation stage, cells undergo mesenchymal-to-epithelial (MET) transition, characterized by the repression of transforming factor beta (TGFbeta), activation of bone morphogenic protein (BMP), stress activated of mitogen-activated protein kinase (MAPK) and p38 MAPK signaling, and extensive remodeling of chromatin (Maherali and Hochedlinger, 2009; Li et al., 2010; Chen et al., 2011; Hu et al., 2014; Neganova et al., 2016; Neganova et al., 2017; Francesconi et al., 2019; Meir and Li, 2021). A number of epigenetic regulators of chromatin, including DOT1L methyltransferase, histone demethylase LSD1, CBP/EP300 bromodomains, bromodomain-containing protein BRD9, histone chaperone CAF-1, BET family proteins, RNA Pol II regulator RPAP1, SUMO modification, chromatin regulator FACT, histone deacetylases HDACs, methyltransferase Setdb1, and the TF TRIM28, act as potent barriers to reprogramming (Cheloufi et al., 2015; Wei et al., 2015; Shao et al., 2016; Sun et al., 2016; Miles et al., 2017; Cossec et al., 2018; Kolundzic et al., 2018; Lynch et al., 2018; Ebrahimi et al., 2019; Sevinc et al., 2022). Some of these factors maintain somatic cell gene expression programs, and some suppress the MET transition, mainly during the initial stage of reprogramming. Inhibition of these regulators greatly enhances the reprogramming of various types of cells. The DNA methylation epigenetic regulators Tet1/2 demethylases, poly (ADP-ribose) polymerase-1 Parp1, and cytidine deaminase AID are critical in reactivating pluripotency genes during this stage of reprogramming (Bhutani et al., 2010; Doege et al., 2012; Hu et al., 2014). After completing MET, cells increase their proliferation rate, suppressing proapoptotic and senescence factors, which are required for extensive epigenetic modification (Marion et al., 2009a; Hong et al., 2009; Utikal et al., 2009; Yunusova et al., 2017). MET also leads to the upregulation of genes involved in cell proliferation, metabolism, and cytoskeletal organization—c-Myc, MycN, KLF4, and Pdzk1 (Prieto et al., 2021). At this stage, cells lack DNA methylation, and active histone modifications mediate the suppression of cell type-specific genes (Polo et al., 2012).

At the beginning of the maturation phase, induction of certain pluripotency genes, such as Oct4, Sox2, Fbxo15, Nanog, Foxh1, and Sall4, takes place (Samavarchi-Tehrani et al., 2010; Buganim et al., 2012; Hansson et al., 2012; Polo et al., 2012; Takahashi et al., 2014). Not all pluripotency genes are activated during this phase, indicating the importance of tight and sequential regulation of events during this stage. During the late phase of maturation, exogenic transgenic constructs are silenced, which is followed by activation of the pluripotency-controlling gene network via 1) modification of the DNA methylation pattern by the DNA methyltransferases DNMT1 and DNMT3 and 2) modification of histones by histone methyltransferase G9a, histone demethylase Utx/Kdm6a, and histone deacetylase HDAC2 (Stadtfeld et al., 2008a; Brambrink et al., 2008; Mikkelsen et al., 2008; Sommer et al., 2009; Bhutani et al., 2010; Mali et al., 2010; Samavarchi-Tehrani et al., 2010; Golipour et al., 2012; Mansour et al., 2012; Polo et al., 2012; Nissenbaum et al., 2013; Wei et al., 2015). This stage represents a prolonged, gradual gene reactivation process associated with the recruitment of the polycomb group and NuRD complex, as well as the upregulation of genes involved in the regulation of chromosomal segregation and cytoskeletal organization (Mansour et al., 2012; Chen et al., 2013; Luo et al., 2013; Rais et al., 2013). By the beginning of the stabilization phase, the activation of additional pluripotent genes, such as *Sox2*, *Utf1*, *Lin28a*, *Esrrb*, *Klf5*, *Dppa2*, *and Dppa4*, as well as extensive epigenetic modifications, occurs (Maherali et al., 2007; Okita et al., 2007; Wernig et al., 2007; Brambrink et al., 2008; Stadtfeld et al., 2008b; Buganim et al., 2012). The processes of extensive reconfiguration of DNA methylation during this stage participate in the reactivation of the X chromosome, recovery of telomere size, and stabilization of the pluripotent epigenetic landscape (Stadtfeld et al., 2008b; Epsztejn-Litman et al., 2008; Mikkelsen et al., 2008; Marion et al., 2009b; Polo et al., 2012; Soufi et al., 2012; Huang et al., 2015; Bar et al., 2019).

## Metabolic features of pluripotent stem cells, in brief

Research on ECSs and iPSCs, collectively called pluripotent stem cells (PSCs), relies predominantly on *in vitro and ex vivo* cell culture-based conditions that are rather different from those provided by native niches. The cell culture conditions include significantly modified metabolic parameters, such as unlimited metabolite supply from the cell culture medium and a lack of hypoxia conditions, both of which differ from *in vivo* ICM surroundings. The first feature of iPSCs and ESCs growing under culture conditions is a high metabolic flux via glycolysis (Cho et al., 2006; Kondoh et al., 2007; Folmes et al., 2011). PSCs utilize glycolysis as a main source of adenosine triphosphate (ATP) production, generating a large amount of lactate (Xu et al., 2013) and shunting the metabolites through the pentose phosphate pathway (Varum et al., 2011; Rodrigues et al., 2015a; Kim et al., 2015). In contrast, upon ESC differentiation, glycolysis is downregulated, while oxidative phosphorylation becomes a dominant bioenergetic source of ATP (Chung et al., 2010; Prigione et al., 2010). In this regard, PSCs prefer hypoxic conditions, under which they rely mostly on glycolytic metabolism activated by hypoxia-inducible factor 1- and 2-alpha (HIF1/2α) and the glycolytic sensors – C-terminal binding proteins (CTBPs) (Powers et al., 2008; Millman et al., 2009; Forristal et al., 2010; Mohyeldin et al., 2010; Arthur et al., 2019; Nakamura et al., 2021). Consistent with lesser dependence on OxPhos for ATP production, PSCs have fewer mitochondria. Furthermore, these organelles are small, globular-shaped, and immature lamellar cristae; contain low copy numbers of mitochondrial DNA; and are predominantly localized in the perinuclear region—and all these parameters are associated with pluripotency (Baharvand and Matthaei, 2003; Cho et al., 2006; Lonergan et al., 2006; Armstrong et al., 2010; Suhr et al., 2010; Folmes et al., 2011; Varum et al., 2011; Zhou et al., 2012; Ware et al., 2014; St John, 2016). However, PSC-specific mitochondria are fully functional in terms of respiratory and OxPhos activities, and the mitochondrial functions are important for the maintenance of pluripotency (Zhang et al., 2011; Seo et al., 2018). A knockdown of mitochondrial DNA polymerase subunit γ (POLG), affecting mitochondrial gene expression, enhances ESC differentiation (Facucho-Oliveira et al., 2007). Extreme mitochondrial fragmentation and mitophagy impair mitochondrial homeostasis, resulting in ESC differentiation (Todd et al., 2010). If compared with differentiated somatic cells, mitochondria in PSCs have reduced OxPhos capacities (Cho et al., 2006; Kondoh et al., 2007; Folmes et al., 2011). Although the composition of the mitochondrial electron transport chain (ETC) in PSCs is different from that of differentiated cells, it is fully functional; nevertheless, mitochondrial production of ATP is maintained at a suboptimal level in these cells. Presumably, low OxPhos in PSCs is also important to maintain a low level of mitochondrial ROS generation to avoid potential ROS damaging activity toward proteins, lipids, and nucleic acids (Armstrong et al., 2010; Prigione et al., 2010). Due to low OxPhos and tricarboxylic acid (TCA) cycle activity, PSCs generate increased levels of metabolic intermediates, which are exported by mitochondria and utilized for various metabolic and signaling epigenetic purposes, for instance, the biosynthesis of fatty acids or the acetylation of histones (Wellen et al., 2009; Moussaieff et al., 2015; Intlekofer and Finley, 2019). The reduced TCA cycle activity in PSCs is maintained by shunting pyruvate out of mitochondria and, in part, via elevated uncoupling protein 2 (UCP2) and reduced pyruvate dehydrogenase (PDH) activities (Zhang et al., 2011). Several studies have revealed a feature of PSCs to actively maintain high mitochondrial membrane potential (MMP), which is important for maintaining a pluripotent state (Schieke et al., 2008; Armstrong et al., 2010; Prigione et al., 2011). The high MMP is maintained partly by the ATP hydrolase activity of ATP synthase, which utilizes ATP produced by glycolysis (Zhang et al., 2011). The function of high MMP in the maintenance of pluripotency requires further clarification, as it is suggested to participate in maintaining various metabolic processes, including regulation of the mitochondria network, anabolic processes, and certain epigenetic processes (Mattenberger et al., 2003; Shyh-Chang et al., 2011; Folmes et al., 2012; Teslaa and Teitell, 2015).

## Naive and primed pluripotency: mouse and human iPSCs

Pluripotency is featured during mammalian development by the cell of the epiblast for a relatively short time interval between the pre-implantation blastocyst and post-implantation egg cylinder stages. Cultured mouse PSCs derived from pre-implantation epiblasts are represented by ESCs and those from post-implantation epiblast-stage cells are represented by the epiblast stem cells (EpiSCs) (Brons et al., 2007; Tesar et al., 2007). These different developmental states of pluripotency are referred to as naïve and primed, respectively. The developmental and functional differences between these pluripotency states are that primed PSCs have more restricted differentiation potential, they are not able to contribute to germ cells. Under cell culture conditions, mouse (m) and human (h) ESCs are maintained in naïve and primed pluripotency states, respectively (Thomson et al., 1998; Klimanskaya et al., 2006; Nichols and Smith, 2009; Nakamura et al., 2016). The same applies to mouse and human iPSCs, and the process of reprogramming into these cells have marked species-specific differences. In addition, the conversion of hESCs and hiPSCs into a naïve state is still an unresolved task in many aspects (Sagi and Benvenisty, 2016; Weinberger et al., 2016; Yilmaz and Benvenisty, 2019). Comparative transcriptional analysis and other analyses of the naïve and primed hPSCs have shown that these pluripotency states have major differences in the landscapes of transcription factors, chromatin remodeling, activity of signaling pathways, cell surface molecules, X chromosome activity, and transposable elements’ expression (Gafni et al., 2013; Guo et al., 2016; Qin et al., 2016; Theunissen et al., 2016; Collier et al., 2017; Messmer et al., 2019; Yilmaz and Benvenisty, 2019). Global DNA hypomethylation is featured in naive hPSCs, which contrasts with the global DNA hypermethylation signature in primed ones (Theunissen et al., 2016; Messmer et al., 2019).

 Importantly, there are also differences in the preference over metabolic pathways between naive and primed hPSCs. Oxidative phosphorylation dominates and lipogenesis tends to be active in naive hPSCs, while primed hPSCs rely on glycolysis for energy metabolism (Takashima et al., 2014; Cornacchia et al., 2019). Naïve mPSCs have a bivalent metabolism, with both glycolysis and OxPhos being active (Weinberger et al., 2016).

The mouse model of cell reprogramming is the most advanced, and the main molecular data were gained from this system. In this model, mouse cells are reprogrammed into naïve type iPSCs within 12 days, whereas it takes 16–28 days to generate primed hiPSCs. In addition, reprogramming efficiency is much lower for humans than for mouse systems (Takahashi and Yamanaka, 2016). The exact molecular differences between these systems still need to be clarified; however, it is known that, unlike in the mouse model, c-Myc transgene is more critical, and MET is a much later event in the human reprogramming process (Cacchiarelli et al., 2015; Xing et al., 2020; Thangavelu and Norden-Krichmar, 2023). Various molecular differences between these systems have also been observed, including JNK signaling, ERRα/ERRγ, and other activities (Yao et al., 2014; Kida et al., 2015; Neganova et al., 2016).

## Pluripotency TFs in regulation of metabolic genes during reprogramming

**FIGURE 2:**
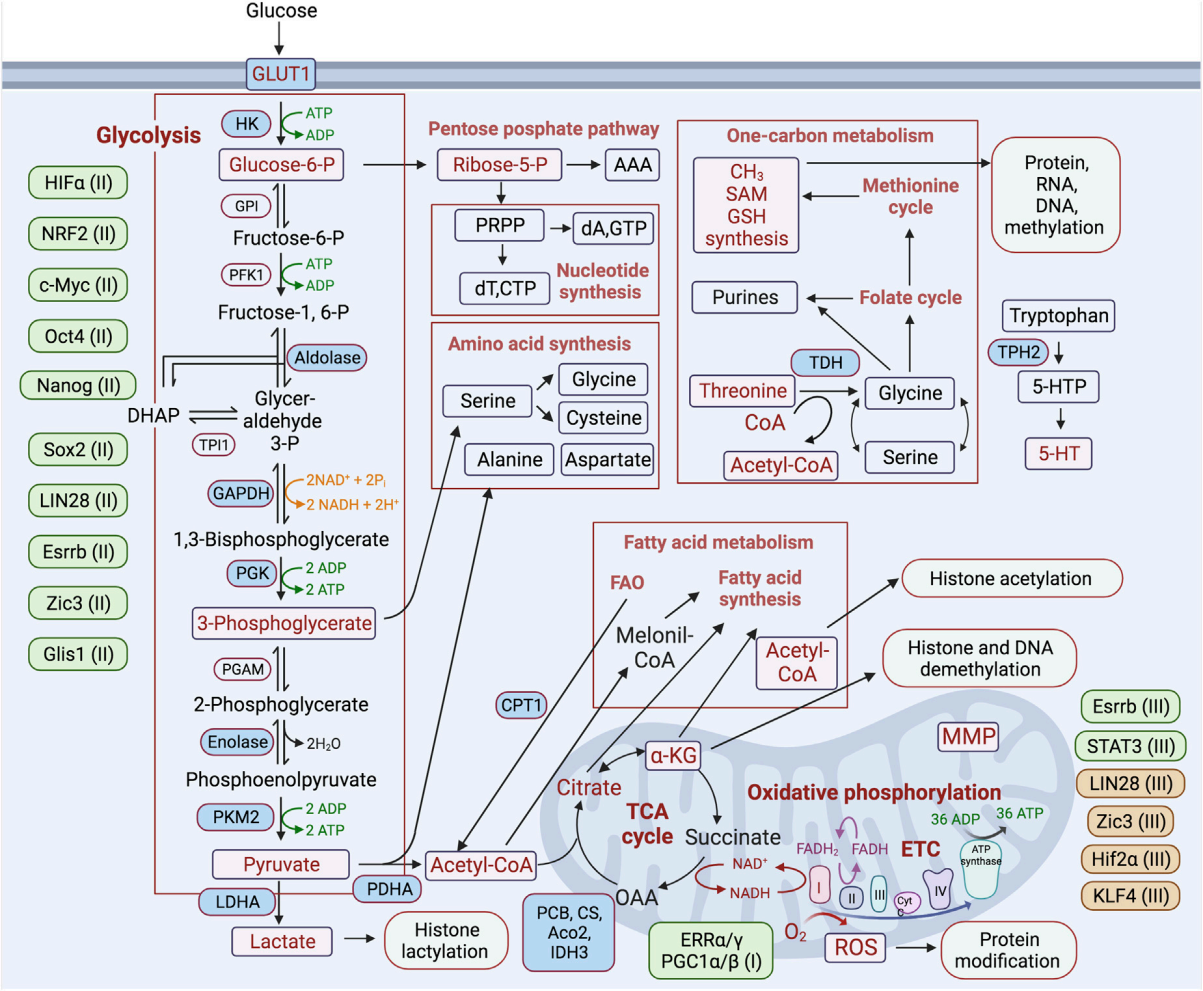
Schematic representation of the major metabolic pathways involved in regulating the process of reprogramming differentiated cells toward a pluripotent state. Metabolic pathways are highlighted in boxes, and key metabolic enzymes and metabolites involved in the regulation of reprogramming are noted in blue and purple rectangles, respectively. Transcription factors participating in positive and negative regulation of certain metabolic pathways are placed next to each of these pathways and depicted in green and brown, respectively. (I-III)—indicated for the initiation (I), maturation (II), stabilization (III) stages of the reprogramming process.

Key pluripotency TFs are involved in the direct regulation of primarily glycolytic genes. The reprogramming factor Oct4 acts in transcriptional regulation of multiple metabolic genes (Kang et al., 2009; Shen et al., 2017). In PSCs, Oct4 directly regulates rate-limiting enzymes of glycolysis—pyruvate kinase M2 (PKM2) and hexokinase 2 (HK2) (Figure 2) (Folmes et al., 2011; Prigione et al., 2014; Kim et al., 2015; Qin et al., 2017). It has also been shown that silencing glucose transporter GLUT3 or PKM2 causes a reduction of OCT4 expression, suggesting a feedback mechanism between glucose metabolism and pluripotent TF gene expression (Christensen et al., 2015). It has been shown that Oct4’s DNA-binding capacity depends on oxidation by ROS. The ROS-scavenging enzyme thioredoxin (Txn), through interaction with cysteines in the POU-domain of Oct4, is able to restore Oct4’s DNA-binding, and furthermore, the gain of Txn function enhances Oct4’s transcriptional activity (Guo et al., 2004). The regulatory sites of the core pluripotent TFs Sox2, Oct4, and Nanog have been identified in an enhancer of the human *GLUT1* gene involved in glycolytic flux in ESCs (Yu et al., 2019). Other studies have shown that chicken *HK1*, *platelet phosphofructokinase* (*PFKP*), and *lactate dehydrogenase A* (*LDHA*) gene promoters contain binding sites for these TFs (Ding et al., 2022). c-MYC also possesses a function to activate glycolysis, which is required to maintain pluripotent status (Varlakhanova et al., 2010; Folmes et al., 2013a; Gu et al., 2016; Cliff et al., 2017; Prieto et al., 2021; Anatskaya and Vinogradov, 2022). c-MYC upregulates expression of PKM2 and LDHA, supporting increased glycolysis (Figure 2) (Cao et al., 2015). These observations are consistent with an activation of the expression of endogenous c-Myc during the initiating stage of reprogramming (Cao et al., 2015). The TF sterol regulatory element binding protein-1 (Srebp-1), involved in lipid homeostasis, enhances reprogramming efficiency through interaction with and strengthening of the function of c-Myc in the expression of pluripotent genes (Wu et al., 2016).

Although the endogenous activation of the pluripotency factor KLF4 occurs during the initiation stage of reprogramming, its function in metabolic regulation has not yet been determined. It has been shown that KLF4 plays an important role in metabolic regulation during the late stage of cell reprogramming (Figure 1). KLF4 positively regulates T cell leukemia/lymphoma protein 1 (TCL1) expression at the late stage of reprogramming (Nishimura et al., 2017; Nishimura et al., 2019). TCL1 suppresses the activity of mitochondrial RNA importer RNAase and polynucleotide phosphorylase (PnPase) through direct interaction with these proteins (Wang et al., 2010). It has been suggested that KLF4-induced TCL1 expression reduces mitochondrial content and OxPhos during the late stage of reprogramming (Khaw et al., 2015; Nishimura et al., 2017). The RNA-binding protein LIN28a/b, which is a critical factor in cell reprogramming and pluripotency maintenance, functions predominantly during the late/stabilization phase and is an important regulator of metabolic genes, mediating activation of glycolysis and maintaining low mitochondrial function, nucleotide, and one-carbon metabolisms (Shyh-Chang et al., 2013a; Zhang et al., 2016; Mathieu and Ruohola-Baker, 2016; Pieknell et al., 2022).

## TFs’ regulation of metabolic pathways during the early/initiation stage of reprogramming—the OxPhos burst stage

Estrogen-related nuclear receptors (ERRs) and their partners—peroxisome proliferator-activated receptor gamma coactivator 1-a/and b (PGC-1α/β)—are transiently expressed at an early stage of reprogramming. ERRα or ERRγ functions, respectively, mediate the burst of OxPhos at the early stage of iPSC generation in the human and mouse systems (Figures 1, 2) (Kida et al., 2015). This metabolic burst occurs upon the activation of glycolysis and OxPhos genes, including mitochondrial ATP synthase (ATP5G1), succinate dehydrogenase (SDHB), isocitrate dehydrogenase (IDH1/3A), and NADH dehydrogenase (NDUFA2), while blocking this metabolic change impedes the reprogramming process. It has been shown that in mice, ERRγ and PGC-1β are highly expressed in Sca1 (−)/CD34 (−) *bona fide* iPSC progenitors or intermediate cells, in which a high level of OxPhos is maintained (Kida et al., 2015).

Nuclear factor (erythroid-derived 2)-like 2 (NRF2) is a major TF involved in the regulation of antioxidant responses. The early burst in OxPhos and consequent ROS generation leads to an increase in NRF2 activity, which promotes a subsequent glycolytic shift through HIF1α activation after the early stage of reprogramming (Hawkins et al., 2016). It has been shown that hypoxic conditions or HIF1α stabilization enhance reprogramming efficiency (Yoshida et al., 2009; Mathieu et al., 2014; Ishida et al., 2020). At this early stage of reprogramming, HIF1α and HIF2α are stabilized, mediating the metabolic shift by activating the expression of glycolysis genes, such as pyruvate dehydrogenase kinase 3 (PDK3), GLUT1/3, LDHA, and HK2. However, only HIF2α stabilization at the late stage of reprogramming has been found to cause major suppression of iPSC generation, in part through activation of tumor necrosis factor-related apoptosis-inducing ligand (TRAIL)-mediated apoptosis (Mathieu et al., 2014). This indicates at least two functions of HIFs during cell reprogramming. Krüppel-like zinc finger TF Glis1 mediates epigenetic and metabolic remodeling and is able to reprogram senescent cells into a pluripotent state, improving genome stability. During the initiation stage of reprogramming, Glis1 upregulates glycolysis by opening chromatin in glycolytic gene loci and closing chromatin in somatic ones (Figure 2) (Maekawa et al., 2011; Li et al., 2020).

Activity of the tumor suppressor p53, as well as inhibited cell proliferation, hinder the acquisition of pluripotency (Marion et al., 2009a; Hong et al., 2009; Kawamura et al., 2009; Utikal et al., 2009). It has been shown that NAD-dependent deacetylase sirtuin-1 (SIRT1) is activated and functions during the initiation phase of reprogramming by acting, in part, through deacetylation of p53, inhibition of p21, and enhancement of Nanog expression. This SIRT1 activity is suppressed through miR-34a, leading to higher p53 activity and, therefore, reduced reprogramming efficiency (Lee et al., 2012). The forkhead box TFs Foxd1 and Foxo1, participating in the regulation of ROS and cellular metabolism, may also be involved in oxidative burst regulation during reprogramming, as their knockdown suppresses the generation of iPSCs (Koga et al., 2014).

## TFs during the late-reprogramming phase

Another ERR-family TF, ERRβ/Esrrb, and the zinc finger of the cerebellum 3 (Zic3) regulate metabolic fluxes synergistically and variably, leading to enhanced reprogramming efficiency. These 2 TFs activate many glycolytic genes cooperatively, resulting in increased glucose consumption. At the same time, unlike Zic3, Esrrb activates OxPhos and oxygen consumption by inducing genes of mitochondrial complex IV (CIV), triggering the morphological remodeling of mitochondria during cell reprogramming (Sone et al., 2017). The ectopic expression of both Esrrb and Zic3 during reprogramming significantly enhances the efficiency of iPSC generation (Figures 1, 2). On the other hand, blocking both glycolysis and OxPhos lowers reprogramming efficiency (Sone et al., 2017). These functions of Esrrb and Zic3 appear to be engaged at the late stage of reprogramming, suggesting that Esrrb may be involved in completing the final epigenetic chromatin modifications specific for naïve pluripotency. This conclusion is supported by the finding that Esrrb can replace Nanog function in late iPSC precursors (Festuccia et al., 2012). In addition, it has been shown that Esrrb reactivation in these cells is dependent on ascorbic acid (Tran et al., 2015), suggesting that this event is a rate-limiting step during the late stage of reprogramming.

It has been shown that activating the Janus kinase/signal transducer and activator of the transcription 3 (Jak/Stat3) pathway is essential for reprogramming. Inhibiting Jak/Stat3 activity blocks demethylation of Oct4 and Nanog regulatory elements during the late reprogramming phase, affecting epigenetic modification and retroviral transgene silencing. Jak/Stat3 activity plays an important role in promoting the establishment of pluripotency during the late phase of reprogramming at the epigenetic level by facilitating DNA demethylation and open chromatin formation in pluripotent loci, including Oct4, Nanog, and the Dlk1-Dio3 regions (Tang et al., 2012; Wang et al., 2018). It is important to note that during reprogramming from the primed to naive states of pluripotency, Stat3 upregulates mitochondria-encoded transcripts and facilitates an increase in mitochondrial metabolism (Carbognin et al., 2016). This suggests that Stat3 activity is required during the late stage of reprogramming, facilitating the optimal maintenance of mitochondrial respiration and epigenetic modifications, both of which are dependent on TCA cycle metabolites.

## Metabolic fluxes during cell reprogramming

### Glycolysis

Glycolysis is a catalytic pathway that converts glucose molecules, through a series of redox reactions, into two pyruvate molecules, generating two ATP and two reduced nicotinamide adenine dinucleotide (NADH) molecules. This pathway does not require oxygen but allows efficient ATP production when glucose is abundant, maintaining the ATP/ADP ratios required for highly proliferative cells. However, glycolysis is much less energetically efficient than the complete oxidation of pyruvate in the TCA cycle and in OxPhos. Nevertheless, highly proliferative cell types, such as cancer and PSCs, predominantly exploit glycolysis (aerobic glycolysis) despite an excess of OxPhos-supportive oxygen. The main reason for the switch to glycolysis is incomplete glucose oxidation, which enables the use of pyruvate for both anabolic needs and the biosynthesis of macromolecules supporting biomass production in proliferating cells (Vander Heiden et al., 2009). Intermediates of the glycolytic pathway are also used by different anabolic pathways: (1) lipid synthesis via acetyl-Coenzyme A (acetyl-Co A), (2) dihydroxyacetone phosphate (DHAP), nucleotide, and NADPH synthesis through glucose-6-phosphate and the pentose phosphate pathways (Figure 2) (Gu et al., 2016; Lees et al., 2018; Lees et al., 2019). It has also been suggested that the dominance of glycolysis over OxPhos is accompanied by reduced ROS generation, which may be protective for the cell due to the preservation of its genomic integrity. The reduction of both OxPhos and its byproduct ROS is less damaging to nuclear and mitochondrial DNA and RNA, as well as to proteins and lipids (Perales-Clemente et al., 2014). The metabolic switch from OxPhos to glycolysis after hyperenergetic flux at the early stage of cell reprograming is a crucial step in iPSC generation. Induction and maintenance of high levels of glycolytic activity during cell reprogramming precede the induction of pluripotency markers and are critical for the successful accomplishment of this process (Folmes et al., 2011; Cao et al., 2015). Activation of 3′-phosphoinositide-dependent kinase-1 (PDK1) by the PS48 compound, which stimulates AKT serine/threonine-protein kinase signaling, in combination with the ectopic expression of Oct4, is sufficient to reprogram cells to a pluripotent state (Zhu et al., 2010). These studies have also shown that AKT activation correlates with increased expression of glycolysis-related genes and increased lactate production. It has been shown that direct or indirect stimulation of glycolysis with D-fructose-6-phosphate or with an activator of HIF1α, ethyl 3,4-dihydroxybenzoate (EDHB), enhances reprogramming, while inhibition of glycolysis with UCN-01, 2-deoxyglucose (2DG), or 3-bromopyruvic acid (BrPA) suppresses reprogramming (Sato et al., 2002; Yoshida et al., 2009; Zhu et al., 2010; Folmes et al., 2011; Panopoulos et al., 2012; Prigione et al., 2014). Accordingly, cells with a dominance of glycolytic over OxPhos gene expression patterns can be reprogrammed more efficiently (Panopoulos et al., 2012). Upon inactivation of the TCA cycle and increased glycolysis after the early stage of reprogramming, lactate is increasingly produced and becomes actively involved in the modification of histones and other targets with the recently identified posttranslational modification—lactylation (Zhang et al., 2019; Li et al., 2020). Activation of glycolytic flux by the TF Glis1 increases acetyl-CoA and lactate levels, thereby enhancing H3 histone acetylation (H3K27Ac) and lactylation (H3K18la) within the chromatin of pluripotency genes, leading to their activation at the intermediate stage of reprogramming (Li et al., 2020). Taken together, these studies suggest that the switch to glycolytic metabolism is a driving force on the route to pluripotency (Figure 2) (Zhu et al., 2010; Folmes et al., 2011).

### Mitochondria functions and remodeling during cell reprogramming

Mitochondria are complex multifunctional organelles and act as the main metabolic hub maintaining cellular homeostasis due to their major role as energy generators, as well as regulators of ROS production, NAD+/NADH balance, calcium homeostasis, signal transduction, and synthesis of most metabolites, including fatty acids, amino acids, iron/sulfur clusters, pyrimidines, heme, and steroid hormones (Dimmer and Scorrano, 2006; Labbe et al., 2014; Picard and Shirihai, 2022; Zhu et al., 2022; Quintana-Cabrera and Scorrano, 2023). Mitochondria are also involved in the regulation and remodeling of cellular processes such as stem cell self-renewal, differentiation, and proliferation (Liesa and Shirihai, 2013; Ma et al., 2015; Folmes et al., 2016; Folmes and Terzic, 2016; Khacho et al., 2016; Matilainen et al., 2017; Seo et al., 2018). The mitochondria of somatic cells serve as the main source of ATP production, representing mature, elongated, branching, filamentous network organelles. In contrast, the mitochondria of PSCs are immature and represent a lower density, possess fragmented morphology, show perinuclear localization, have disordered mitochondrial cristae, and have reduced mtDNA copy numbers (Cho et al., 2006; Lonergan et al., 2006; Suhr et al., 2010; Folmes et al., 2011; St John, 2016). During differentiation, extensive mitochondrial biogenesis and remodeling occurs, resulting in elongated branching filamentous networks. Mitochondrial morphology and dynamics are linked with cristae shape and supercomplex assembly, which are critical parameters directly regulating the main mitochondrial functions—respiratory efficiency, TCA cycle, OxPhos, ROS generation, and redox state (Baker et al., 2019).

It has been shown that during cellular reprogramming, mitochondria are gradually reconstructed to an ESC-specific immature state, characterized by rounded morphology with poor cristae structure, less active mitochondrial respiration, and reduced mtDNA copy number (Armstrong et al., 2010; Prigione et al., 2010; Folmes et al., 2011; Folmes et al., 2012; Choi et al., 2015). A significant re-organization of the mitochondrial network during the transition from somatic to pluripotent states occurs (Seo et al., 2018; Skvortsova et al., 2022). The number of mitochondria dramatically changes in iPSC precursors, reaching its peak at the initiation stage and then declining dramatically during the maturation and stabilization stages of the reprogramming (Figure 1). The decline of mitochondrial content during reprogramming, resulting in fewer spherical mitochondria with poorly developed and immature cristae, is probably mediated by the mitophagy process (Prigione and Adjaye, 2010; Folmes et al., 2011; Pan et al., 2013; Ma et al., 2015; Choi et al., 2015; Naik et al., 2019) and the inhibition of mitochondrial biogenesis (Wang et al., 2020). An early and transient activation of autophagy occurs during reprogramming through downregulation of the mTOR pathway (Wang et al., 2013; Xiang et al., 2017), and overactivation of this pathway during the early stage of reprograming is suppressive for the process (Wu et al., 2015). The activation of autophagy and mitophagy is mediated in part by elevated levels of ROS (Bolisetty and Jaimes, 2013), which may be triggered during the oxidative burst phase of reprogramming. However, the impact of mitophagy and mitochondrial biogenesis at this particular stage of reprogramming requires more investigation. The peak of mitochondrial content coincides with an ERR-mediated oxidative burst and an increase in mitochondrial and Nox-generated ROS, and these events induce NRF2/Hif1-α-triggered activation of glycolytic flux in iPSC precursors after day 3, followed by a subsequent major decline in mitochondrial numbers and activity by the mid- and late-reprogramming stages (see below).

Mitochondrial fusion and fission dynamics are crucial for somatic cell reprogramming. Activation of mitochondrial fragmentation, which is mediated by dynamin-related protein 1 (DRP1), favors the reprogramming process at the initiation stage (Vazquez-Martin et al., 2012a; Prieto et al., 2016). During this stage, Ras/ERK1/2-mediated activation of Drp1 allows its recruitment to mitochondria and triggers mitochondrial fragmentation (Serasinghe et al., 2015; Prieto et al., 2016), which is also associated with the activation of cyclin-dependent kinase 1 (Cdk1) via cyclin B protein level increase (Prieto et al., 2018). It has also been shown that the pluripotency of TF REX1 mediates activation of DRP1/mitochondrial fission and, in parallel, activates cyclin B1/B2 expression (Son et al., 2013). In agreement with this, the activation of mitochondrial fusion by ectopic expression of mitofusin-1 or -2 (MFN1 and MFN2)—proteins that activate mitochondrial fusion—blocks the reprogramming (Figure 1) (Son et al., 2015). MFN1/MFN2 deficiencies increase reprogramming efficiency by participating in the switch to glycolytic metabolism. This process is associated with inhibition of the p53-p21 pathway, activation of RAS/RAF signaling, and ROS-mediated HIF1α stabilization (Son et al., 2015). The endoplasmic reticulum (ER) participates in mitochondrial fission; also, the unfolded protein response (UPR) of mitochondria and ER are activated during cell reprogramming. Transient activation of ectopic UPR, perhaps in concert with mito-fission activation, enhances cell reprogramming (Simic et al., 2019). Furthermore, mitochondria fragmentation is associated with an increased release of mtROS, which also peaks on day 3 of reprogramming (Tang et al., 2009; Bolisetty and Jaimes, 2013; Skvortsova et al., 2022), contributing to the regulation of the oxidative burst-mediated switch to glycolytic metabolism (discussed below). Thus, the processes involved in the activation of mitochondrial fragmentation are associated with stimulation of the cell cycle progression; they all occur during the initiation phase and are crucial factors for efficient cell reprogramming.

### TCA cycle and iPSC reprogramming

In mitochondria, the TCA (or Krebs) cycle represents the main metabolic pathway mediating energy production, as well as supporting the cycle intermediates to support pathways involved in central carbon metabolism and epigenetic remodeling. In this catalytic pathway, pyruvate, which is produced mainly via glycolysis, is oxidized to CO_2_, resulting in the generation of reduced forms of the electron donors (NADH and flavin adenine dinucleotide, FADH2) required for OxPhos and ATP synthesis by donating electrons to the electron transport chain. The TCA cycle mediates the reactions to replenish TCA intermediates, using predominantly pyruvate and glutamine as substrates (Figure 2). The TCA cycle also supplies partially oxidized intermediates as building blocks for anabolic processes, including lipid, amino acid, and nucleotide biosynthesis, as well as for post-translational and epigenetic modifications of proteins and DNA (Wellen et al., 2009; Ruiz et al., 2012; Vardhana et al., 2019). The TCA cycle is critical for the regulation of cellular processes such as stem cell self-renewal, differentiation, and reprogramming through the regulation of energy generation and supply of substrates for anabolism and macromolecule modifications. In contrast to differentiated cells, PSCs feature incomplete oxidation of pyruvate, allowing them to use TCA cycle intermediates for many cellular processes, including anabolic synthetic pathways and histone modification. It has been shown that, in opposition to inactivation, activation of PDH by pyruvate dehydrogenase kinase (PDK) inhibitor dichloroacetate (DCA) reduces reprogramming efficiency, suggesting that activation of the TCA cycle and suppression of glycolysis have a suppressive effect on the process (Folmes et al., 2011; Rodrigues et al., 2015b).

The TCA cycle provides metabolites for epigenetic modifications in the nucleus during embryonic development and pluripotency induction. For example, the excess of TCA cycle intermediates in ESCs promotes the generation of citrate, which supports the additional production of cytoplasmic acetyl-CoA for protein acetylation (Moussaieff et al., 2015). The increased levels of cycle metabolites—α-ketoglutarate (α-KG), S-adenosyl methionine (SAM), malic acid, and cofactors NAD+/NADH—are also important for the regulation of epigenetic and metabolic modifications during the acquisition and maintenance of pluripotency (Figure 2) (Lee et al., 2012; Shiraki et al., 2014; TeSlaa et al., 2016; Tran et al., 2019b; Figlia et al., 2020). α-KG generated by isocitrate dehydrogenase 3 (Idh3) is an important substrate for DNA and histone demethylation (TeSlaa et al., 2016). SAM, derived from one-carbon metabolism, is a methyl donor for both DNA and histone methylation (Shyh-Chang et al., 2013b; Shiraki et al., 2014). Reduced NADH is required for NADH-dehydrogenase CI ETC/OxPhos activity and ATP production (see below) (Skvortsova et al., 2022), whereas NAD+/NADH balance is involved in the regulation of different post-transcription modifications during cell reprogramming (Lee et al., 2012). Recent studies have shown that translocation of TCA cycle enzymes—Pdha1, pyruvate carboxylase (Pcb), aconitase 2 (Aco2), citrate synthase (Cs), and isocitrate dehydrogenase 3 (Idh3a)—to the nucleus promotes reprogramming (Figure 2) (Li et al., 2022). This indicates that non-canonical functions of these enzymes outside of mitochondria reside in the nucleus and are likely to mediate a supply of metabolites for sufficient epigenetic modifications, which are extensively required during reprogramming. For instance, nuclear Pdha1 increases the acetyl-CoA pool in the nucleus, leading to chromatin remodeling within pluripotency genes by enhancing histone H3 acetylation. TCA cycle intermediate metabolites are critical for modifications of the epigenome in iPSCs and, thereby, are involved in the regulation of gene expression and pluripotency acquisition (Carey et al., 2015; Moussaieff et al., 2015; Zhang et al., 2016; Guitart et al., 2017; Zhang et al., 2018; Fang et al., 2019). Nevertheless, further investigations of the involvement of particular metabolites produced by the TCA cycle during the process of iPSC generation are yet required.

Amino acids are substrates for the biosynthesis of proteins, lipids, and nucleotides; they are also involved in *de novo* purine biosynthesis and amino acid metabolism and are linked with one-carbon metabolism (OCM), maintaining cellular pools of one-carbon residues associated with S-adenosylmethionine (SAM) and folate (Locasale, 2013; Shuvalov et al., 2017; Clare et al., 2019). This OCM donates methyl groups for the synthesis of amino acids, nucleotides, and phospholipids; it also supplies substrates, such as SAM, for the post-translational methylation of RNA, DNA, and proteins, linking changes in metabolism to epigenetic remodeling of cells (Figure 2). Threonine metabolism is required to maintain cellular pluripotency. Lack of threonine in cell culture medium or inhibition of the key enzyme of threonine metabolism, threonine dehydrogenase (TDH), results in a loss of self-renewal, apoptosis, and cell cycle arrest in PSCs (Wang et al., 2009; Alexander et al., 2011; Ryu and Han, 2011). Also, L-threonine supplementation and the ectopic expression of TDH enhance the efficiency of cell reprogramming (Han et al., 2013). The threonine metabolism can contribute to this process via several mechanisms, for example, by supplying methyl groups to OCM to reinforce the biosynthesis of cellular building blocks or by supporting SAM to promote the pluripotent state-specific H3K4me3 (Bernstein et al., 2006; Wang et al., 2009; Shyh-Chang et al., 2013b). The biosynthesis of the neurotransmitter serotonin by a rate-limiting enzyme, tryptophan hydroxylase-2 (TPH2), is also involved in the regulation of cell reprogramming. Removing TPH2 function during the entire reprogramming process majorly enhances the efficiency of iPSC generation, while TPH2 induction suppresses the process, indicating the importance of tryptophan pool or serotonin signaling in pluripotency acquisition (Sinenko et al., 2023). The latter possibility is supported by the observed activity of serotonin in the positive regulation of mitochondrial functions and biogenesis (Fanibunda et al., 2019; Rao et al., 2023). It has also been shown that a key enzyme in glycine degradation, glycine decarboxylase (GLDC), is involved in the early stages of cell reprogramming (Kang et al., 2019). GLDC expression appears to be regulated by the reprogramming factors KLF4 and c-MYC, and loss of GLDC activity impairs reprogramming, probably through a reduction in glycolytic intermediates, which suggests that it can function by upregulating glycolysis (Kang et al., 2019).

In addition to playing a structural role as the main components of membranes, lipids are important energy sources and signaling mediators. Lipid metabolism includes *de novo* biosynthesis and catabolism through fatty acid oxidation (FAO); both of these processes occur in equilibrium and are highly context- and cell-dependent. It has been shown that lipogenesis is enhanced during reprogramming, as fatty acid synthase (FASN) and acetyl-CoA carboxylase 1 (ACACA) expression are increased during the process; in addition, supplying extra oleic acid can increase reprogramming efficiency (Wang et al., 2017). ACACA-mediated increase in lipogenesis supported enhanced mitochondrial fission by blocking acetylation-dependent ubiquitin-proteasome degradation of the mitochondrial fission 1 protein. The pharmacological inhibition of ACACA and FASN activities markedly decreases reprogramming efficiency, whereas stimulating the activity of these key lipogenic enzymes promotes this process (Vazquez-Martin et al., 2013; Wu et al., 2016). Ectopic expression of Srebp-1, a transcriptional factor required for lipid homeostasis, was found to enhance reprogramming, while its knockdown suppresses the process (Wu et al., 2016). The inhibition of lipid biosynthesis and autophagosome formation significantly reduce reprogramming efficiency. It has been shown that Rab32 kinase-A anchoring protein increases lipid biosynthesis and storage, as well as autophagosome formation, during the early and middle phases of reprogramming, thus improving its outcome (Pei et al., 2015).

On the other hand, the catabolic FAO process is important at the early stage of reprogramming, which coincides with the abovementioned OxPhos burst. Activation of FAO by adding palmitoylcarnitine—a product of the carnitine palmitoyltransferase (CPT) system—during the first 3 days of reprogramming is sufficient to stimulate OxPhos activity, thereby enhancing reprogramming (Figures 1, 2) (Lin et al., 2018). After this stage, OxPhos is suppressed by palmitoylcarnitine supplementation. CPT1b, which is a rate-limiting enzyme of FAO, is significantly upregulated at the early stage of reprogramming, and its ectopic expression was found to significantly improved reprogramming efficiency. Thus, via different mechanisms, the activation of both lipid anabolism and catabolism support iPSC generation.

### OxPhos and ETC in cell reprogramming

OxPhos is the main bioenergetic pathway operating in mitochondria, which enables the transfer of electrons donated by NADH and FADH_2_ of the TCA cycle to the redox reaction carried out by the ETC complexes. This process creates potential gradients and harnesses the energy to pump protons from the matrix to intramembrane space through the inner mitochondrial membrane, generating an electrochemical proton membrane gradient. Mitochondrial ATP synthase using the membrane gradient transfers protons back across the inner membrane, thereby generating ATP. OxPhos is a far more efficient bioenergetic pathway for ATP production than glycolysis (36 vs. 2 ATP molecules per glucose). Furthermore, OxPhos maintains bioenergetic homeostasis by linking multiple metabolic pathways, including glycolysis, the TCA cycle, and FAO. ETC contains five multi-subunit complexes, I-V (CI-CV). NADH-dehydrogenase CI is the largest 45-subunit complex mediating dehydrogenase activity by accepting electrons from NADH and the main contributor to electrochemical gradient force. CII accepts electrons from FADH_2_ and contributes much less than CI to the total electrochemical force (Figure 2).

It was shown that most subunits of CI, CII, and CIV are downregulated in iPSCs, while subunits of CIII and CV were shown to be upregulated in these cells, compared to the mouse embryonic fibroblasts (MEFs) from which the iPSCs were derived (Folmes et al., 2011). The CI and CII reduction in PSCs suggests that OxPhos is mainly suppressed in these cells, while the glycolytic pathway is dominant. As mentioned above, the proteomic analysis identified dynamic and tightly coordinated changes in expression and in the stoichiometry of ETC complexes at all stages of the reprogramming (Hansson et al., 2012). The decreased expression of NADH-dehydrogenase CI and CIV, along with increased expression of CII, CIII, and CV subunits at the early stage, might reflect possible stabilization of ROS levels during the oxidative burst to avoid an excess of its production (Boekema and Braun, 2007; van Raam et al., 2008). The reduced CI and increased CII presence at the early stage of the reprogramming may suggest that FADH_2_ dominates over NADH in electron donation to ETC, probably to avoid excessive ROS production and to increase the NADH/NAD + ratio, which can suppress the TCA cycle and overall OxPhos activity (Kida et al., 2015; Lee et al., 2016). OxPhos capacity reaches a peak by days 2–3 of reprogramming (Kida et al., 2015). We have shown that CI is important for cell reprogramming. Continuous suppression of CI function through silencing NADH-ubiquinone oxidoreductase subunits S1 or B10 (Ndufs1 or Ndub10) or through rotenone treatment during the entire process of reprogramming impedes iPSC generation. In contrast to the first 3 days of reprogramming, functioning CI is in greater demand during the intermediate and late stages of the process in terms of reprogramming efficiency, suggesting that efficient OxPhos is required during the entire process of iPSC generation (Skvortsova et al., 2022). In agreement with this observation, it has been demonstrated that induced mitochondrial respiratory dysfunction caused by pathogenic heteroplasmy of mitochondrial DNA (mtDNA), which encodes ETC complex subunits, also suppresses reprogramming (Hamalainen et al., 2015; Yokota et al., 2015). However, rare heteroplasmic iPSC clones derived via this reprogramming acquired proper pluripotency status. It has yet to be elucidated which part of OxPhos and ETC complexes is required for each stage of cell reprogramming (Figure 1). Among redox signaling regulation by mitochondrial ROS (Skvortsova et al., 2022), OxPhos can be important for TCA cycle metabolite supply, NADH and Acetyl CoA balance, MMP maintenance, and ATP production during each phase of reprogramming. The contribution of CI to ATP generation and MMP maintenance at various stages of cell reprogramming requires investigation. Cells with inhibited ETC are not able to oxidize TCA cycle-produced NADH, resulting in the suppression of this catalytic cycle. This may result in diminished biosynthesis of citrate and its derivative, cytosolic acetyl-CoA, and, therefore, in reduced histone acetylation (Martinez-Reyes et al., 2016). While this issue requires further investigation, it seems likely that immortalized somatic cells or cancer cells already recruit aerobic glycolysis and suppress OxPhos metabolic flux. These cells are unlikely to achieve an oxidative burst during the onset of reprogramming, which may explain their inability to achieve a pluripotent state (Vazquez-Martin et al., 2012b; Lee et al., 2016; Park et al., 2017; Skvortsova et al., 2018).

### ROS signaling during cell reprogramming to pluripotency

ROS are several partially reduced (gaining electrons) oxygen-containing molecules, including superoxide anion (O_2_^−^), hydrogen peroxide (H_2_O_2_), and hydroxide radicals, all of which are highly poised to interact with and modify various types of biological molecules. High and continuous ROS exposure under, for example, conditions when the cell antioxidant system is deficient causes oxidative damage, leading to cell and mitochondrial genome failure, thereby promoting cell senescence and death (Halliwell and Gutteridge, 1984; Papa and Skulachev, 1997; Holzerova and Prokisch, 2015). However, it is well established that ROS function as important intracellular signaling molecules that operate through post-translational covalent modifications of various signaling proteins, as well as through specific sensors that acutely respond to redox-based intracellular changes (Hamanaka and Chandel, 2010; Suda et al., 2011; Autreaux and Toledano, 2007; Sinenko et al., 2021b; Sies and Jones, 2020; Andreyev et al., 2005; Lyublinskaya et al., 2015; Sauer et al., 2001). Physiological ROS signals, mediated mainly by hydrogen peroxide, are also important for stem cell functions, promoting the proliferation and differentiation of these cells (Le Belle et al., 2011; Suda et al., 2011; Sinenko et al., 2021b; Ivanova et al., 2021). ROS can be produced in the cytosol mainly by plasma membrane NAD(P)H oxidases (NOXs) and in mitochondria by ETC complexes. Alongside several enzymes of the TCA-cycle–2-oxoglutarate dehydrogenase, monoamine oxidases (MAOs), 2-oxoacid dehydrogenase complexes (OADHC), mitochondrial snglycerol-3-phosphate dehydrogenase (mGPDH), and dihydroorotate dehydrogenase (DHDOH)—the electron-transferring flavoprotein (ETF/ETF:QOR system) contributes to ROS generation in mitochondria (Brand, 2016). In addition, in mitochondria, electron leakage occurs due to electron transport ETC function, leading to the formation of O_2_^−^, H_2_O_2_, and other ROS-related molecules (Korshunov et al., 1997; Wong et al., 2017). The main sources of ROS in ETC are complexes I and III (Andreyev et al., 2005; Chernyak et al., 2006; Murphy, 2009; Vinogradov and Grivennikova, 2016; Chenna et al., 2022), and it was shown that CI contributes mainly to O_2_^−^ generation under physiological conditions (Votyakova and Reynolds, 2001; Kushnareva et al., 2002). CI inactivation by genetic or pharmacological means leads to significantly increased ROS levels (sublethal levels), which mediate different physiological processes in various model organisms (Sinenko et al., 2012; Tan et al., 2017; Sinenko et al., 2021b; Knapp-Wilson et al., 2021; Skvortsova et al., 2022). Multilevel antioxidant defense and regulatory systems tightly regulate ROS levels in a cell type- and cell compartment-dependent manner, mediated by redox-controlled TFs and antioxidant enzyme systems, such as superoxide dismutase-1, -2 (SOD-1/2), catalase, and peroxiredoxins 1-6 (Pxn1-6) (Andreyev et al., 2005; Sies and Jones, 2020; Sinenko et al., 2021b).

The tight regulation of ROS levels is also required for different stages of cell reprogramming to pluripotency (Figure 1). It has been shown that activation of the innate immunity Toll-like receptor 3 (TLR3) pathway triggers ROS generation and signaling at the onset of the reprogramming, which is required for its efficient accomplishment (Lee et al., 2012; Yang et al., 2013). The NADPH oxidase 2 (NOX2) enzyme complex is involved in the generation of ROS at this stage of reprogramming, and optimal levels of ROS signaling are essential to induce pluripotency (Zhou et al., 2016). Along with NOX-generated ROS, mitochondria-derived ROS (mtROS) contribute greatly to ROS signaling at the early stage of reprogramming (Hawkins et al., 2016; Skvortsova et al., 2022). As previously mentioned, levels of mtROS peak on day 3 of the process, coincident with an OxPhos burst and high mitochondrial content. At this stage, the main ROS contributors, CI and CIII, are correspondingly down- and upregulated, probably to attenuate the overproduction of ROS. This idea is consistent with the observation that removing excess ROS during the early reprogramming phase significantly increases the efficiency of iPSC generation (Skvortsova et al., 2022). On the other hand, cells undergoing reprogramming at this stage are the most resilient to OxPhos inactivation and high ROS levels, as they better tolerate loss of CI activity compared with later stages of the reprogramming. The exact mechanisms by which reprogramming is regulated by ROS and antioxidant response systems requires further investigation.

The abundant ROS generated by OxPhos and NOX2 during the early reprogramming period are likely to be critical for driving metabolic transition during subsequent phases of reprogramming. This might be mediated by NRF2 activation via the modification of cysteine residues of the NRF2 repressor KEAP1, leading to HIF1α activation and stimulation of glycolysis (Mathieu et al., 2014; Hawkins et al., 2016). It has also been shown that elevated ROS can stabilize HIF1α, promoting reprogramming (Chandel et al., 2000; Son et al., 2015). The increased ROS might also act via nuclear factor kappa B (NF-κB) and c-Jun/activator protein-1 (AP-1) by inactivating these TF, as their inactivation increases the efficiency of iPSC generation (Filosto et al., 2003; Lingappan, 2018; Markov et al., 2021). The high ROS levels can potentially signal through the p38 MAPK and JNK pathways to mediate cell reprogramming processes during the early stages (Son et al., 2013; Neganova et al., 2016; Neganova et al., 2017). The function of ROS to support the proliferation of various cells, including PSCs (Ivanova et al., 2021; Kirova et al., 2022) and cells during reprogramming (Zhou et al., 2016; Skvortsova et al., 2022), is consistent with the importance of active proliferation at the early stage of reprogramming. It has been shown that Nrf2 action during reprogramming is also mediated by increased proteasome activity, particularly by proteasome maturation protein (POMP) (Buckley et al., 2012; Jang et al., 2014; Selenina et al., 2017; Zubarev et al., 2022), which probably acts through the degradation of metabolism-related proteins.

The impact of the significantly reduced number of mitochondria and decreased mtROS at the mid- and late stages of reprogramming requires better understanding. OxPhos suppression and increased ROS generation upon CI inactivation at these stages result in significantly reduced reprogramming efficiency, while maintaining certain levels of ROS at these stages is supportive of the reprogramming process (Skvortsova et al., 2022). Intriguingly, optimal ROS levels during reprogramming are crucial for the process, as simultaneous CI inhibition by rotenone and ROS scavenging by N-acetyl cysteine synergistically suppress reprogramming (Skvortsova et al., 2022). However, during these stages, increased ROS presumably has a damaging proapoptotic effect, and a certain counterbalancing by antioxidant activity is required to improve the quality of human iPSCs (Armstrong et al., 2010; Ji et al., 2014). In this regard, it is necessary to keep an optimal ROS level during every stage of the reprogramming, and the corresponding molecular mechanisms governing these processes await further clarification.

### Mitochondria genome and reprogramming

The mitochondrial genome and functions are well preserved throughout the processes of reprogramming peripheral blood mononuclear cells to iPSCs and during differentiation of the latter cells into functional cerebral organoids (Duong et al., 2021). Several studies have investigated the effects of mitochondrial respiratory dysfunction triggered by mutant mtDNA on cellular reprogramming. The variable amount of mutant mtDNA in somatic cells allows for the generation of patient-specific iPSC lines with high or low heteroplasmic levels during reprogramming procedures (Ma et al., 2015; Kodaira et al., 2015). Further, when iPSC lines are established, mtDNA heteroplasmy levels are not significantly altered during *in vitro* differentiation, so higher and lower percentages of heteroplasmy are preserved in fully differentiated cells (Folmes et al., 2013b; Klein Gunnewiek et al., 2020; Duong et al., 2021).

The generation of patient-derived iPSCs and derivatives thereof, carrying various heteroplasmic mtDNA mutations, has also been reported (Fujikura et al., 2012; Folmes et al., 2013b; Cherry et al., 2013; Hamalainen et al., 2013; Yokota et al., 2015). Patient-specific and cell-type-specific variation of the molecular pathogenic potential of mutant m.3243A>G mtDNA was demonstrated (Yokota et al., 2015). In addition, *in vitro* recapitulation of neuronal pathophysiology in mitochondrial encephalopathy, lactic acidosis, and stroke-like episodes (MELAS) was performed using iPSC-derived neurons (Hamalainen et al., 2013). Thus, the listed data demonstrate that iPSC-based approaches are highly useful for modeling and investigating mtDNA disease progression and the phenotypes of affected tissues.

A high-depth mtDNA sequence analysis of human iPSCs and primary fibroblasts confirmed the age-related accumulation of mtDNA mutations in human fibroblasts. However, this analysis showed that heteroplasmy levels vary during cell reprogramming and shape the mtDNA landscape on a clonal level, resulting in the heterogeneity of derived iPSC lines (Wei et al., 2021). On the other hand, heteroplasmy levels remained stable during the cell differentiation process. It was also shown that during iPSC culturing, there is a strong functional selection against mutations in mitochondrial genes that encode ETC complex subunits (Hamalainen et al., 2015; Kosanke et al., 2021). Thus, from a mtDNA perspective, reprogramming to pluripotency and iPSC culturing clears or “rejuvenates” the cell population by removing cells carrying mutated mtDNA, which resembles the processes that occur during germline reprogramming. In the female germline, rapid shifts in heteroplasmy due to various modes of mtDNA content reduction have been shown (Cree et al., 2008; Wai et al., 2008; Cao et al., 2009; Burr et al., 2018). A similar “bottleneck” effect can also occur at the cellular level due to the preferential replication and loss of cells containing specific mtDNA variants during reprogramming. mtDNA variations have been shown to influence reprogramming efficiency (Latorre-Pellicer et al., 2019).

## Conclusion

Since the discovery of TF-mediated pluripotency reprogramming, an extensive pool of data has been developed, helping to understand the molecular mechanisms of the reprogramming process. In particular, during the last decade, significant insight into the role of metabolic regulation in stem cell pluripotency acquisition has been obtained. It was established that metabolic demands in differentiated cells and PSCs are different. The metabolism of slow-proliferating differentiated cells is dependent mainly on OxPhos and active catabolic pathways, while the metabolism of highly proliferative PSCs is dependent mainly on the activation of aerobic glycolysis and anabolic pathways. The reprogramming of differentiated cells into PCSs requires a major transformation of the energy metabolism. The most critical metabolic change occurs during the early stage of reprogramming, with the initiation of the switch from OxPhos to aerobic glycolysis dominancy. At this stage, an OxPhos burst that includes an increase in respiratory mitochondrial parameters occurs, resulting in a burst of ATP and ROS production. These events are likely to represent a signal to activate NRF2/HIF1a and other transcriptional regulators and to induce and maintain the activation of glycolytic and anabolic pathways. They also induce significant changes in the mitochondrial network, which is gradually transformed into an immature state through fragmentation and mitophagy processes. While the dominancy of the glycolytic pathway during the maturation and stabilization stages of reprogramming is established, the OxPhos function and associated optimal levels of ROS, ATP, and MMP production are required for reprogramming process. It has been shown that metabolic changes often determine and instruct changes during cell fate transitions and reprogramming. This can be mediated through interaction with signaling pathways and TF networks of various metabolites, including NAD+, ATP, ROS, and retrograde signals from the main metabolic organelles, mitochondria. In addition, the contribution of metabolic pathway metabolites to the epigenetic remodeling of the cell takes place. Recent studies have also shown that a single metabolic intermediate from active metabolic pathways—glycolysis, TCA cycle, FAO, and fatty acid synthesis—can dramatically affect stem cell identity and the reprogramming process.

Single-cell resolution analysis of gene expression during the reprogramming process identified that, along with the relatively minor population of true iPSC progenitor cells that become iPSCs, many cells pass different non-pluripotent cell fates. In most reprogramming studies, these populations were not considered in terms of their impact on metabolic processes and possible cell non-autonomous effects. These processes, along with many questions about the metabolic regulation of reprogramming, are only beginning to be deciphered and still require clarification using, for example, single-cell-based metabolomics methodology.

iPSCs are the most promising cell source for cell-based therapies in regenerative medicine. In light of this, it is especially important to gain a better understanding of molecular mechanisms, metabolic regulations, and epigenetic signatures involved in the reprogramming process to improve the timing and quality of the generated iPSCs and develop better cell sources for regenerative medicine.

## Figures and Tables

**Table:** 

**Acetyl-CoA**	Acetyl-Coenzyme A
**AAA**	Aromatic amino acids
**AMPK**	AMP-activated protein kinase
**AP-1**	Activator protein 1
**ATP**	Adenosine three phosphate
**α-KG**	alpha-ketoglutarate
**BMP**	Bone morphogenetic protein
**CI-IV**	Complexes I-IV
**CMA**	Cell-matrix adhesion
**CPT**	Carnitine palmitoyltransferase
**CTBP**	C-terminal binding protein
**DHAP**	Dihydroxyacetone phosphate
**DRP1**	Dynamin-related 1
**ERK**	Extracellular signal-related kinase
**ERRα/γ**	Estrogen-related nuclear receptor alpha/gamma
**ESRRB**	Estrogen-related nuclear receptor beta
**ESCs**	Embryonic stem cell
**ETC**	Electron transport chain
**GLDC**	Glycine decarboxylase
**GLUT1/3**	Glucose transporter 1/or 3
**FADH2**	Flavin adenine dinucleotide
**FAO**	Fatty acid oxidation
**FASN**	Fatty acid synthase
**FIS1**	Mitochondrial fission 1 protein
**HIF1/or 2α**	Hypoxia-inducible factor 1/or 2-alpha
**HK1/or 2**	Hexokinase 1/or 2
**ICM IDH1/3**	Isocitrate dehydrogenase 1/or 3
**iPSCs**	induced pluripotent stem cells
**KEAP1**	Kelch like-ECH-associated protein 1
**LIF**	Leukemia inhibitory factor
**LDHA**	Lactate dehydrogenase A
**MAPK**	Mitogen-activated protein kinase
**MEFs**	Mouse embryonic fibroblasts
**MET**	Mesenchymal-to-epithelial transition
**MELAS**	Mitochondrial encephalopathy, lactic acidosis, and stroke-like episodes
**MMP**	Mitochondrial membrane potential
**mtDNA**	Mitochondrial DNA
**mTOR**	Mammalian target of rapamycin
**MFN1/2**	Mitofusin-1/or 2
**NADH**	Nicotinamide adenine dinucleotide
**NADPH**	Nicotinamide adenine dinucleotide phosphate
**NDUFS1 NADH**	ubiquinone oxidoreductase core subunit S1
**NF-kB**	Nuclear factor kappa B
**NRF2**	Nuclear factor (erythroid-derived 2)-like-2
**NOX2**	NADPH oxidase 2
**OCM**	One-carbon metabolism
**OSKM**	OCT4, SOX2, KLF4, and cMYC
**OxPhos**	Oxidative phosphorylation
**PDH**	Pyruvate dehydrogenase
**PDHK1**	Pyruvate dehydrogenase kinase 1
**PDK1**	3-Phosphoinositide-dependent protein kinase-1
**PDK3**	Pyruvate dehydrogenase kinase 3
**PGC-1**	Peroxisome proliferator-activated receptor-1
**PKM2**	Pyruvate kinase M2
**PN TFs**	Pluripotency network TFs
**PnPase**	Polynucleotide phosphorylase
**POLG DNA**	polymerase subunit
**POMP**	Proteasome maturation protein
**PRPP**	Phosphoribosyl pyrophosphate
**PSCs**	Pluripotent stem cells
**PTBP1**	Polypyrimidine tract-binding protein 1
**ROS**	Reactive oxygen species
**SAM**	S-adenosyl methionine
**SDHB**	Succinate dehydrogenase
**SOD-1/2**	Superoxide dismutase 1/or 2
**TCA**	Tricarboxylic acid
**TCL1**	T cell leukemia/lymphoma protein 1
**TDH**	Threonine dehydrogenase
**TFs**	Transcription factors
**TLR3**	Tall-like receptor 3
**TGFbeta**	Transforming factor beta
**UCP2**	Uncoupling protein 2
**UPR**	Unfolded protein response
